# Phenotypic and genotypic characterization of Shiga toxin-producing *Escherichia coli* strains recovered from bovine carcasses in Uruguay

**DOI:** 10.3389/fmicb.2023.1130170

**Published:** 2023-03-06

**Authors:** Paula Mussio, Inés Martínez, Santiago Luzardo, Armando Navarro, Gerardo Leotta, Gustavo Varela

**Affiliations:** ^1^Departamento de Microbiología, Laboratorio Tecnológico del Uruguay, Montevideo, Uruguay; ^2^Latitud, Fundación LATU, Montevideo, Uruguay; ^3^Instituto Nacional de Investigación Agropecuaria, INIA, Tacuarembó, Uruguay; ^4^Departamento de Salud Pública, Facultad de Medicina, Universidad Nacional Autónoma de México, Mexico City, Mexico; ^5^Instituto de Ciencia y Tecnología de Sistemas Alimentarios Sustentables, UEDD INTA-CONICET, Buenos Aires, Argentina; ^6^Departamento de Bacteriología y Virología, Facultad de Medicina, Universidad de la República, Montevideo, Uruguay

**Keywords:** STEC, bovine carcasses, virulence factors, WGS, Uruguay

## Abstract

**Introduction:**

Shiga toxin-producing *Escherichia coli* (STEC) is a zoonotic pathogen that cause food-borne diseases in humans. Cattle and derived foodstuffs play a known role as reservoir and vehicles, respectively. In Uruguay, information about the characteristics of circulating STEC in meat productive chain is scarce. The aim was to characterize STEC strains recovered from 800 bovine carcasses of different slaughterhouses.

**Methods:**

To characterize STEC strains we use classical microbiological procedures, Whole Genome Sequencing (WGS) and FAO/WHO risk criteria.

**Results:**

We analyzed 39 STEC isolated from 20 establishments. They belonged to 21 different O-groups and 13 different H-types. Only one O157:H7 strain was characterized and the serotypes O130:H11(6), O174:H28(5), and O22:H8(5) prevailed. One strain showed resistance *in vitro* to tetracycline and genes for doxycycline, sulfonamide, streptomycin and fosfomycin resistance were detected. Thirty-three strains (84.6%) carried the subtypes Stx2a, Stx2c, or Stx2d. The gene eae was detected only in two strains (O157:H7, O182:H25). The most prevalent virulence genes found were *lpfA* (*n* = 38), *ompA* (*n* = 39), *ompT* (*n* = 39), *iss* (*n* = 38), and *terC* (*n* = 39). Within the set of STEC analyzed, the majority (81.5%) belonged to FAO/WHO’s risk classification levels 4 and 5 (lower risk). Besides, we detected STEC serotypes O22:H8, O113:H21, O130:H11, and O174:H21 belonged to level risk 2 associate with diarrhea, hemorrhagic colitis or Hemolytic-Uremic Syndrome (HUS). The only O157:H7 strain analyzed belonged to ST11. Thirty-eight isolates belonged to the Clermont type B1, while the O157:H7 was classified as E.

**Discussion:**

The analyzed STEC showed high genomic diversity and harbor several genetic determinants associated with virulence, underlining the important role of WGS for a complete typing. In this set we did not detect non-O157 STEC previously isolated from local HUS cases. However, when interpreting this findings, the low number of isolates analyzed and some methodological limitations must be taken into account. Obtained data suggest that cattle constitute a local reservoir of non-O157 serotypes associated with severe diseases. Other studies are needed to assess the role of the local meat chain in the spread of STEC, especially those associated with severe diseases in humans.

## 1. Introduction

Shiga toxin-producing *Escherichia coli* (STEC) includes more than 1,000 different serotypes defined according to somatic “O” and flagellar “H” antigens combinations. The set of these serotypes associated with severe diseases in humans is classically called enterohemorrhagic *E. coli* (EHEC) (Croxen et al., 2013; FAO and WHO, 2019; Malahlela et al., 2022).

The human illnesses that STEC produce range from mild pathologies like watery diarrhea (WD), severe bloody diarrhea (BD) to life-threatening entities such as hemorrhagic colitis (HC) and hemolytic-uremic syndrome (HUS). The latter is defined by the presence of thrombocytopenia, mechanical hemolytic anemia and multi-organ ischemic damage (Rivas et al., 2016).

STEC strains can also be grouped into two serological categories, STEC O157 and STEC non-O157. *Escherichia coli* O157:H7 is the leading serotype of STEC isolated clinically, and generally produce more severe illnesses than non-O157:H7 STEC ones. In the last decade, non-O157 STEC strains with atypical virulent characteristics (e.g., *eae* negative) linked to cases of severe human diseases have been recovered worldwide. The main non-O157 serogroups in Europe include O26, O80, and O145, while in United States O26, O45, O103, and O111 are the most common [Hadler et al., 2011; EFSA and ECDC (European Food Safety Authority and European Centre for Disease Prevention and Control), 2022].

The global impact of STEC infection has been estimated in 43.1 acute cases per 100,000 persons, with 3,890 annual cases of HUS and more than 100 deaths (Majowicz et al., 2014). Centers for Disease Control and Prevention (CDC) reported that the incidence of STEC infection was 5.9 per 100,000 persons during 2018, a 26% increase over the incidence from 2015 to 2017 (Tack et al., 2019). New Zealand communicated a mean annual incidence of 0.8 per 100,000 persons (2.6/100,000 children under 5 years), France 0.49 per 100,000 persons (3.1/100.000 children under 5 years) and China 0.57 per 100,000 persons [0.38/100.000 children under 5 years; Vally et al., 2012; EFSA and ECDC (European Food Safety Authority and European Centre for Disease Prevention and Control), 2019; Feng et al., 2022]. On the other hand, Latin America has an endemic STEC infection, with most cases located in the south of the continent. In Uruguay it is estimated that occur between 10 and 15 HUS cases per year, and the incidence rate would be 0.5/100.000 inhabitants and 4 to 5/100,000 children under 5 years old (Varela et al., 2008). In neighboring Argentina, which has a robust surveillance system, HUS incidence during 2021 was 0.6/100.000 people and 5.95/100,000 children under 5 years of age, with a lethality of 1.7% (BEN 611-SE29 – Boletín epidemiológico nacional N611 SE29, 2022). It has been estimated that STEC infections in South America cause approximately 2% of acute diarrhea cases and 20–30% of BD (Torres et al., 2018).

As a result of their genetic plasticity, STEC of the same serotype may have different virulence profiles and represent different human health risks. This characteristic can also lead to the emergence of hybrid strains. In that sense, in 2011, a hybrid EAEC-STEC O104:H4 caused an important outbreak in Europe. Between 1992 and 2012, four EAEC-STEC hybrid strains were associated with small outbreak and sporadic cases of HUS (Koutsoumanis et al., 2020; Torti et al., 2021). This situation highlights the need to advance in the analysis and estimation of the virulent potential of local STEC strains (Rasko et al., 2011; Robins-Browne et al., 2016).

The ability of STEC to cause damage has been classically related to their capacity to produce different variants of the Shiga toxin (Stx). These variants are classified into two types: Stx1 (which consists of three variants Stx1a, Stx1c, and Stx1d) and Stx2 (that included variants Stx2a, Stx2b, Stx2c, Stx2d, Stx2e, Stx2f, and Stx2g). Stx1a, Stx2a, Stx2c, and Stx2d are linked to severe human diseases but there is no definitive or conclusive association, since it has been seen that the type of phage that contains the *stx* gene, the site where it was inserted and the combination of other genes can affect the virulence of STEC (Scheutz et al., 2012).

It has been postulated that without adherence of STEC to the intestinal epithelium, Stx production alone is considered insufficient to cause serious diseases. Therefore, Stx production and adhesion capacity would be important attributes to determining the course of STEC infections. The major adherence factor is intimin encoded by the *eae* gene, which is located in a pathogenicity island called LEE (Locus of Enterocyte Effacement). However, there are severe disease case reports caused by LEE-negative STEC (Newton, 2009). Thus, it is also interesting to evaluate STEC’s ability to produce human damage the presence of genes linked to other adhesion mechanisms: *aggR*, *aaiC*, *saa*, *sab*, *paa*, *efa*1, *omp*A, *ompT*, *lpfA*, *toxB*, the LAA island (Locus of Adhesion and Autoaggregation) using the *hes* gene as marker, as well as other virulence factors associated with adaptation and toxicity (Paton and Paton, 2002; Kaper et al., 2004; Herold et al., 2009; Montero et al., 2017).

Cattle is the principal reservoir of STEC strains (both, O157 and non-O157 ones), along with other ruminants such as sheep and goats, and from them STEC could be transmitted directly to humans, or through of foods, included meat and milk, or water contaminated with their feces (Farrokh et al., 2013; Menge, 2020). The presence of STEC in meat for human consumption is considered a hazard and has two clearly defined negative impacts. One, the most important, on human health and the other economically related to the losses determined by this production chain, especially in countries like Uruguay, where the majority of meat production is exported to increasingly demanding markets.

As far as we know, up to now there are no many local reports about the presence of STEC in bovine carcasses nor of its microbiological characteristics analyzed using WGS.

The aim of the study was to characterize phenotypically and genotypically a set of STEC strains recovered from bovine carcasses to have an initial approximation to knowledge of the circulating strains.

## 2. Materials and methods

### 2.1. Detection and isolation of STEC

For 2 years (August 2018–July 2020), covering the four seasons, samples of randomly selected half-carcasses of 37 abattoirs from all over the country (13 for internal supply and 24 for export; representing 88% of those enabled for bovine slaughter in Uruguay in 2018) were taken 24 h after sacrifice, with or without intervention (steam vacuum, water washing by arc with sprinklers, application of lactic acid 2–4% at room temperature), being cooled to 4°C. The number of samples per establishment was proportional to their participation in the 2017-year national slaughter, from a minimum of 1 sample to maximum 73. Between 1 and 7 abattoir visits were made.

Carcass swabs were obtained, following the methodology previously described by Brusa in 2017 (Brusa et al., 2017). Briefly, the total surface of the half-carcasses comprising both external and internal face, was covered with a pre moistened sponge (Whirl-Pak, Merck, Germany).

Stomacher bags containing the sponges were placed into coolers with ice and sent to be processed within 18 h. Upon arrival at the laboratory, 100 ml of pre-warmed modified Tryptone Soy Broth (mTSB; Neogen, United States) were added to the sponges and incubated at 42 ± 1°C for 18–22 h. All samples were analyzed by real time PCR using the BAX® System kit “STEC Screening assay for *stx* and *eae*” genes (Hygiena, United States).

If a positive signal for *stx* was detected samples (both, *eae* positive or *eae* negative) were streaked onto two MacConkey (MAC; Oxoid, United Kingdom) plates and, subsequently, in two consecutive eosin-methylene blue–Levine (EMB-Lev, Oxoid, United Kingdom) plates and incubated 37 ± 1°C for 18 h. The confluent growth zones were screened for the *stx1*, *stx2*, and *eae* genes by endpoint multiplex PCR (Paton and Paton, 1998). If the *stx1* or *stx2* genes were detected, up to 50 colonies per sample were selected for PCR confirmation.

Those samples that were simultaneously positive for both *stx* and *eae* genes, were also screened for the presence of *E. coli* O157:H7 using the BAX® System kit “Real time *E. coli* O157:H7” (Hygiena, United States). Positive samples were processed by immunomagnetic separation with Dynabeads® anti-*E. coli* O157 (Thermo Fisher Scientific, United States) following manufacturer’s instructions, and plated onto Sorbitol MAC agar supplemented with cefixime–potassium tellurite (CT-SMAC, Oxoid, United Kingdom) and onto CHROMagar™ O157 (Chromagar, France). Typical sorbitol negative colonies and mauve colored colonies, respectively, were agglutinated using the “*E.coli* O157 Latex Test Kit “(Thermo Scientific, United States).

Presumptive STEC strains were isolated on Trypticase soy agar (TSA, Oxoid, United Kingdom), and kept in 1 ml Trypticase soy broth (TSB, Oxoid, United Kingdom) with 0.5 ml of glycerol at-80°C for further phenotypic and genotypic characterization.

### 2.2. Phenotypic identification, antimicrobial susceptibility tests, and serotyping of STEC strains

Confirmation of isolates as *E. coli* was performed using the “NCCombo 66 panel” on the MicroScan autoSCAN-4 system (Beckman Coulter Inc., United States), according to the manufacturer’s instructions. “Neg MIC 44” panel was also used to determine the Minimum Inhibitory Concentration (MIC) to 33 antimicrobials (Beckman Coulter Inc, 2021). Disk diffusion susceptibility tests were made following the recommendations for *Enterobacteriaceae* of the Clinical and Laboratory Standards Institute (CLSI, 2019). The following 14 antibiotics were tested: ampicillin 10 μg (AMP10); cefuroxime 30 μg (CXM30); ceftriaxone 30 μg (CRO30); fosfomycin/trometamol 200 μg (FOT200); ceftazidime 30 μg (CAZ30); cefepime 30 μg (FEP30); imipenem 10 μg (IPM10); amoxicillin-clavulanic acid 30 μg (AMC30); cefoxitin 30 μg (FOX 30); trimethoprim-sulfamethoxazole 25 μg (SXT25); ciprofloxacin 5 μg (CIP 5); amikacin 30 μg (AK30); gentamicin 10 μg (CN10); and meropenem 10 μg (MEM10) (Oxoid, United Kingdom). Result interpretation was done according to CLSI breakpoints (Clinical and Laboratory Standards Institute, 2019). *Escherichia coli* ATCC 25922 was used as quality control.

Serotypes were determined at the Universidad Nacional Autónoma de México, using classical Ørskov and Ørskov′s agglutination assay with rabbit serum (SERUNAM, Mexico) obtained against 187 somatic antigens and 53 flagellar antigens of *E. coli* (Peirano et al., 2018).

### 2.3. Whole genome sequencing and genotypic characterization

The strains to be studied were thawed and inoculated into TSA agar plates. After an overnight incubation at 37 ± 1°C, five morphologically identical colonies of the pure culture were selected and genomic DNA was extracted and then purified using the DNeasy Blood & Tissue Kit (QIAGEN, Germany), following the manufacturer’s instructions. Genomic libraries were created with the TruSeq Nano DNA Kit – 350pb library (Illumina, United States) and sequencing was performed using the Illumina NovaSeq 150PE platform at Macrogen Inc. (Seoul, South Korea). The accession numbers to the sequences are available in Supplementary Table S1. Read quality was analyzed with FASTQC (Andrews, 2010) and low-quality positions were removed using Sickle (Joshi and Fass, 2011). Subsequently, the reads are assembled into contigs using Velvet in two steps; first velveth converts the reads into k-mers using a hash table, and secondly velvetg assembles the overlapping k-mers into contigs through a Bruijn graph discarding contigs with a length below the 250 bp (Zerbino and Birney, 2008). The web tools of the Center for Genomic Epidemiology (CGE) were used to evaluate the *in silico* molecular characterization of the sequenced strains. The multilocus sequence type was determined using MLST Finder 2.0 against the *E. coli* #1 set, including adenylate kinase (*adk*), fumarate hydratase (*fumC*), DNA gyrase (*gyrB*), isocitrate/isopropylmalate dehydrogenase (*icd*), malate dehydrogenase (*mdh*), adenylosuccinate dehydrogenase (*purA*), and ATP/GTP binding motif (*recA*) genes (Larsen et al., 2012). Virulence Finder 2.0 was used to search for virulence genes and ResFinder 4.1 was used to identify the AMR genes, setting an identity threshold of 90% with a minimum length protein of 60% (Camacho et al., 2009; Joensen et al., 2014; Zankari et al., 2017; Bortolaia et al., 2020). The *stx* subtyping and the following additional virulence factors were searched by *in silico* PCR, using IPCress (Slater and Birney, 2005) and the primers described in Table 1 for: Efa1 adherence factor (*efa1*), LAA Hemagglutinin (*hes*), outer membrane protein A (*ompA*), STEC autoagglutination adhesin (*saa*), an autotransporter that contributes to biofilm formation (*sab*), and an urease-associated protein (*ureC*).

**Table 1:** PCR primers used for the detection of STEC virulence or adherence genes.

Target	Primer	Oligonucleotide sequence (5′–3′)	Amplicon size (bp)	References
*efa1*	efa1F	GAGACTGCCAGAGAAAG	479	Bai et al. (2013)
	efa1R	GGTATTGTTGCATGTTCAG		
*hes*	hes_det1	CAACCAGCGTCTTATCGAT	350	Montero et al. (2017)
	hes_det2	CGGTTGTTTTCTGGTGAAC		
*ompA*	ompAF	TTTTGGATGATAACGAGG	1,156	Wang et al. (2010)
	ompAR	TGCTGGGTAAGGAATAAC		
*saa*	SAADF	CGTGATGAACAGGCTATTGC	119	Paton and Paton (2002)
	SAADR	ATGGACATGCCTGTGGCAAC		
*sab*	LH0147-f	GGTGGATACAGCAGGTAATG	163	Herold et al. (2009)
	LH0147-r	TATCTCACCACCTGCTATCG		
*ureC*	ureCF	TCTAACGCCACAACCTGTAC	397	Franz et al. (2015)
	ureCR	GAGGAAGGCAGAATATTGGG		

Molecular serotyping was made with SerotypeFinder 2.0 using an identity threshold of 85% with a minimum length of 60% (Joensen et al., 2015).

Genomic sequences were also analyzed to determine phylogroups with ClermonTyping (Beghain et al., 2018; Clermont et al., 2019). Finally, the strains were classified with the FAO/WHO criteria to evaluate their potential risk (World Health Organization and Food and Agriculture Organization of the United Nations, 2018).

## 3. Results

### 3.1. Presence of *stx* genes in carcasses and selected STEC strains for a detailed analysis

**Figure 1:**
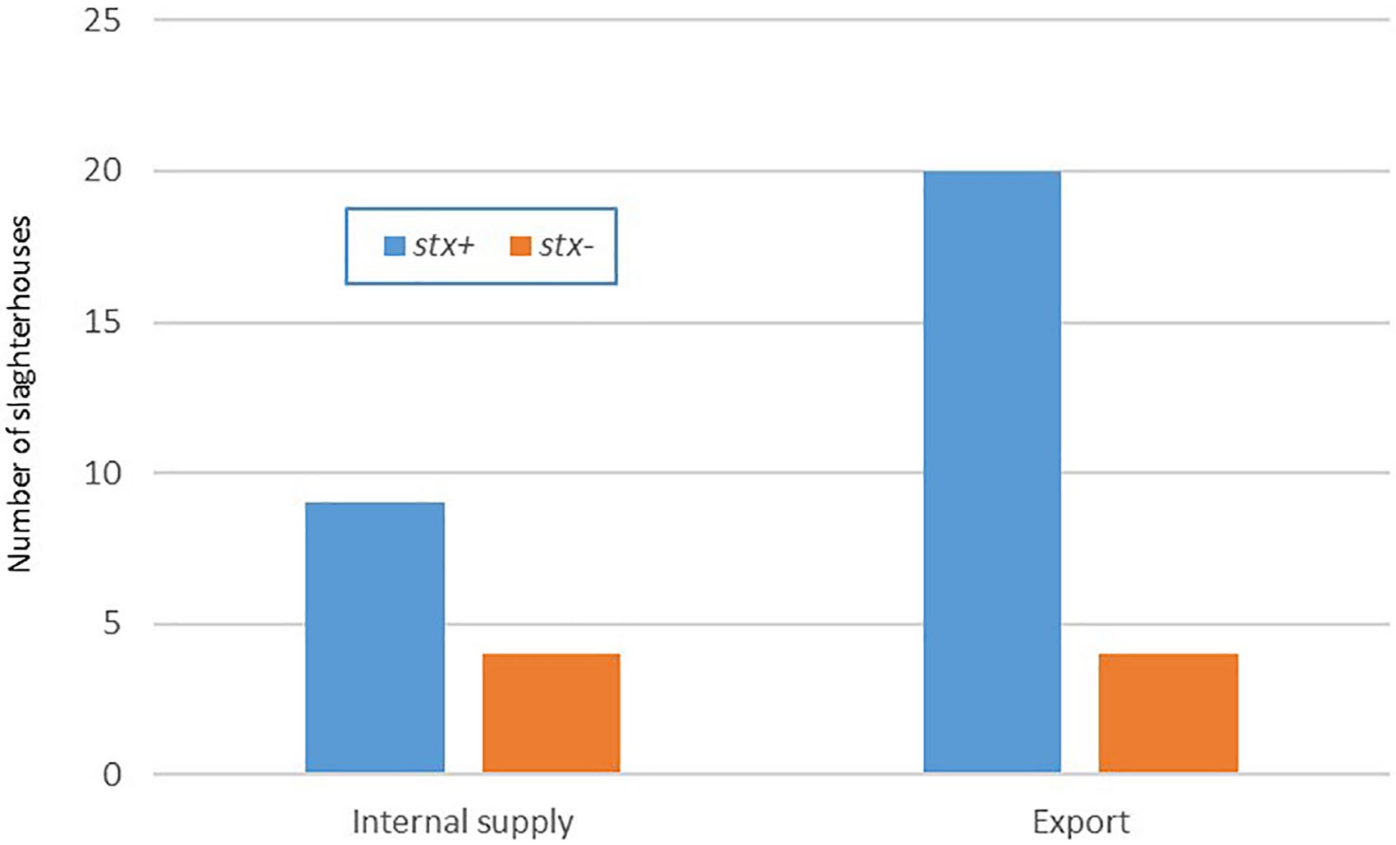
Positive *stx* samples by type of slaughterhouse. Uruguay 2018–2020.

From the 800 carcasses analyzed, 179 (22.3%, CI 95 19.5–25.3%) were positive to *stx* genes, representing 29 of the 37 (78.4%) slaughterhouses sampled 20 for export and nine for local supply (Figure 1). In 25 of them, at least one isolate was recovered. To continue with the characterization analysis, STEC strains from all the positive slaughterhouses were selected taking into account the number of *stx* positive samples from the screening of each establishment. However, in 5 of the them with positive isolates in the initial stage, it was not possible to continue with the characterization since when the strains were thawed they did not present a positive *stx* signal again. Of the 800 samples analyzed, only in 2 we recovered *E. coli* O157:H7 (prevalence 0.25%, CI 95 0–0.6%). Since both isolates came from to the same establishment, with the same sampling date and the same virulence profile (*stx2*c/*eae*), we opted for the WGS characterization of only one of the strains. All the 39 selected strains were identified as *Escherichia coli* using the MicroScan panels. Three isolates were unable to use sorbitol: U20 (O157:H7), U9 (O174:H21), and U22 (O-:H21).

### 3.2. Serotyping using classical procedure and serotype finder database

Serotyping of the isolates showed that the 39 WGS confirmed STEC strains belonged to 20 different O-groups and 13 different H types were identified, as well as one strain that was nontypeable (O-) (Table 2). Only one O157:H7 strain was included and among the 38 non-O157 isolates, the serotypes O130:H11 (6), O174:H28(5), and O22:H8 (5) prevailed. As can be seen in Table 2, there are some strains that could not be serotyped or gave inconsistent results. The isolates O182:H25 (U5), O88:H25 (U6), and O20:H7 (U32) were serotyped by Serotype Finder. Two isolates defined by agglutination test as O156 (U8, U27) could not be identified as such by the Serotype Finder. Some discrepancies could also be observed in the results obtained when assigning the serotype by agglutination or when doing so using the Serotype Finder. Strain U31 belonged to O159:H28 by agglutination and was molecularly identified as O130:H11, also the strain U39 was found to be O99:H19 by serology and was O10:H42 by aligning sequences with CGE databases.

**Table 2:** Characteristics of STEC strains isolated in Uruguay between 2018 and 2020 from bovine carcasses.

Id	Serotype	*stx1*	*stx*2	*eae*	Virulence genotype	MLST *E. col*i#1	Clermont type	Resistance profile	From Abattoir No. (type)	Interventions (Y/N)
U20	**O157:H7**	*stx*1a	*stx*2c	+	*astA, cba, chuA, efa1, espA, espB, espF, espJ, gad, iha, iss, nleA, nleB, nleC, ompA, ompT, terC, tir, traT*	11	E	S	17 (int)	N
U18	O174:H28	−	*stx*2a	−	*cdtB, cea, celb, cia, cib, cvaC, ehxA, epeA, espP, gad, hes, hra, iss, lpfA, ompA, ompT, saa, subA, terC, traT*	156	B1	S	16 (int)	Y
U26	O174:H28	*stx*1a	*stx*2a	−	*cia, espP, gad, hes, hra, iha, iss, lpfA, ompA, ompT, saa, sab, terC, traT*	156	B1	S	19 (int)	N
U50	O174:H28	*stx*1a	*stx*2a	−	*cea, cia, cib, cvaC, ehxA, epeA, espP, gad, hes, hra, iha, iss, lpfA, ompA, ompT, saa, sab, subA, terC, traT*	156	B1	S	2 (exp)	N
U45	O178:H19	−	*stx*2c	−	*espP, gad, hes, hra, iha, iss, lpfA, ompA, ompT, terC, traT*	192	B1	S	13 (exp)	N
U1	**O130:H11**	−	*stx*2a	−	*cba, cea, celb, cia, cib, cvaC, ehxA, epeA, espP, gad, hes, hra, iss, lpfA, mchF, ompA, ompT, saa, subA, terC, traT*	297	B1	S	17 (int)	N
U11	**O130:H11**	*stx*1a	*stx*2a	−	*cea, celb, cia, cib, cvaC, ehxA, epeA, espP, gad, iha, iss, lpfA, ompA, ompT, saa, sab, subA, terC, traT*	297	B1	S	18 (exp)	Y
U14	**O130:H11**	*stx*1a	*stx*2a	−	*cea, celb, cia, cib, cvaC, ehxA, epeA, espP, gad, iha, iss, lpfA, ompA, ompT, saa, sab, subA, terC, traT*	297	B1	S	20 (int)	nd
U23	**O130:H11**	*stx*1a	*stx*2a	−	*cea, celb, cia, cib, cvaC, ehxA, epeA, espP, gad, iha, iss, lpfA, ompA, ompT, saa, subA, terC, traT*	297	B1	S	1 (exp)	N
U37	**O130:H11**	*stx*1a	*stx*2d	−	*cea, cia, cib, cvaC, ehxA, epeA, espP, gad, iha, iss, lpfA, ompA, ompT, saa, sab, subA, terC, traT*	297	B1	S	20 (int)	nd
U49	**O130:H11**	*stx*1a	*stx*2d	−	*cea, celb, cia, epeA, espP, gad, iha, iss, lpfA, ompA, ompT, saa, sab, subA, terC, traT*	297	B1	S	14 (int)	N
U5	O182:H25(a)	*stx*1a	−	+	*astA, ehxA, espA, espJ, espP, etpD, iss, lpfA, nleA, nleB, nleC, ompA, ompT, terC, tir, traT*	300	B1	S	8 (exp)	N
U47	O171:H2	−	*stx*2a,b	−	*cba, gad, hes, hra, iha, iss, lpfA, neuC, ompA, ompT, terC, traT*	332	B1	S	24 (exp)	N
U27	O156:H10(b)	*stx*1d	−	−	*astA, cdtB, cea, cia, gad, lpfA, ompA, ompT, papC, terC*	441	B1	S	25 (exp)	Y
U17	O178:H19	*stx*1a	*stx*2a	−	*cia, ehxA, espP, gad, hes, hra, iha, iss, lpfA, ompA, ompT, saa, sab, subA, terC, traT*	443	B1	S	23 (exp)	N
U3	**O22:H8**	−	*stx*2b,c	−	*espl, gad, iha, ireA, iss, lpfA, ompA, ompT, terC*	446	B1	S	4 (exp)	Y
U4	**O22:H8**	−	*stx*2c	−	*celb, gad, hes, hra, iha, iss, lpfA, ompA, ompT, terC, traT*	446	B1	S	17 (int)	N
U30	**O22:H8**	−	*stx*2d	−	*espl, gad, iha, ireA, iss, lpfA, ompA, ompT, terC*	446	B1	S	13 (exp)	Y
U41	**O22:H8**	−	*stx*2d	−	*espI, gad, iha, ireA, iss, lpfA, ompA, ompT, terC*	446	B1	S	18 (exp)	N
U44	**O22:H8**	−	*stx*2a	−	*gad, hes, hra, iha, iss, lpfA, ompA, ompT, terC, traT*	446	B1	S	9 (exp)	N
U9	**O174:H21**	−	*stx*2d	−	*gad, hes, hra, iha, iss, lpfA, ompA, ompT, terC, traT*	677	B1	S	5 (exp)	N
U10	**O174:H21**	−	*stx*2d	−	*gad, hes, hra, iha, iss, lpfA, ompA, ompT, terC, traT*	677	B1	S	2 (exp)	N
U13	O74:H42	*stx*1a	*stx*2c	−	*cia, ehxA, espP, gad, hes, hra, iss, lpfA, ompA, ompT, saa, terC, traT*	1,172	B1	S	4 (exp)	Y
U22	O-:H21	*stx*1a	*stx*2a	−	*cea, celb, cib, cvaC, ehxA, epeA, gad, iha, iss, lpfA, ompA, ompT, saa, subA, terC, traT*	1,248	B1	S	13 (exp)	N
U6	O88:H25(a)	*stx*1d	−	−	*cia, gad, iss, lpfA, ompA, ompT, papC, terC, traT*	1,679	B1	S	24 (exp)	Y
U19	O120:H7	−	*stx*2c	−	*cia, gad, hes, hra, iha, iss, lpfA, ompA, ompT, terC, traT*	1727	B1	S	8 (exp)	Y
U24	O185:H7	−	*stx*2c	−	*cia, espP, gad, hes, hra, iha, iss, lpfA, ompA, ompT, terC, traT*	2,387	B1	S	11 (int)	N
U25	O185:H7	−	*stx*2c	−	*cia, espP, gad, her, hra, iha, iss, lpfA, ompA, ompT, terC, traT*	2,387	B1	S	14 (int)	N
U46	O116:H49	−	*stx*2a	−	*celb, cia, ehxA, epeA, espP, hes, hra, iha, iss, lpfA, ompA, ompT, saa, subA, terC, traT*	2,520	B1	S	3 (exp)	Y
U34	O8:H16	*stx*1a	*stx*2a	−	*celb, cia, ehxA, espP, gad, hes, hra, iha, iss, lpfA, ompA, ompT, saa, terC, traT*	2,602	B1	S	9 (exp)	N
U42	O8:H16	*stx*1a	−	−	*celb, cia, ehxA, espP, gad, iha, iss, lpfA, ompA, ompT, saa, terC, traT*	2,602	B1	S	12 (exp)	N
U2	**O113:H21**	*stx*1a	*stx*2d	−	*astA, espl, gad, iha, ireA, iss, lpfA, mchB, mchC, mcmA, ompA, ompT, terC*	3,695	B1	S	3 (exp)	Y
U8	O156:H10(b)	*stx*1d	−	−	*cdtB, cia, gad, iss, lpfA, ompA, ompT, terC, traT*	6,190	B1	S	4 (exp)	Y
U15	O8:H19	*stx*1a	*stx*2a	−	*celb, cia, ehxA, espP, gad, iha, iss, lpfA, ompA, ompT, saa, terC, traT*	6,661	B1	S	5 (exp)	Y
U48	O8:H19	*stx*1a	*stx*2a	−	*cba, celb, cia, ehxA, espP, gad, iha, iss, lpfA, ompA, ompT, saa, terC, traT*	6,661	B1	S	21 (int)	N
U38	O6:H34	−	*stx*2c	−	*espI, gad, hra, iha, iss, lpfA, ompA, ompT, terC*	7,616	B1	S	9 (exp)	N
U31	O159:H28(c)	*stx*1a	*stx*2a	−	*cea, celb, cia, cib, cvaC, ehxA, epeA, espP, gad, iha, iss, lpfA, ompT, saa, subA, subA, terC, traT*	ND	B1	S	4 (exp)	Y
U32	O20:H7(a)	−	*stx*2c	−	*espP, hes, hra, iha, iss, lpfA, ompA, ompT, terC, traT*	ND	B1	S	3 (exp)	Y
U39	O99:H19(d)	*stx*1d	−	−	*cdtB, etsC, gad, hra, iss, lpfA, ompA, ompT, terC, traT*	ND	B1	R	13 (exp)	N

(a) Only by molecular serotyping. (b) Not possible to serotype by molecular. (c) Does not match genomic serotyping giving O130:H11. (d) Does not match genomic serotyping giving O10:H42. Serotypes associate with HUS (in bold). nd, Not determined; int, internal supply; exp, exportation; Y, yes, applied mitigation measures; N, did not apply mitigation measures; S Susceptible to: ampicillin; cefuroxime; ceftriaxone; fosfomycin/trometamol; ceftazidime; cefepime; imipenem; amoxicillin-clavulanic acid; cefoxitin; trimethoprim-sulfamethoxazole; ciprofloxacin; amikacin; gentamicin; meropenem; tetracycline and minocycline. R, Resistat to tetracycline and minocycline.

### 3.3. Antibiotic susceptibility profiles and resistance genes

None of the 39 STEC strains analyzed showed resistance to the antimicrobials tested by disk diffusion (see the list of antibiotics tested by this procedure above). In one isolate O120:H7, the fosfomycin resistance gene *fosA*7 was detected with 94.1% of identity but no phenotypic resistance to this antibiotic was expressed. One O99:H19 strain had the resistance genes for: doxycycline, tetracycline, minocycline – *tetB* (100% identity), sulfonamides – *sul2* (100% id) and streptomycin – *aph(3″)-Ib* and *aph(6)-Id* (100% id). Resistance to tetracycline (TE) and minocycline (MIN) was confirmed by Microscan showing MICs values >8 μg/ml to both. Resistance to doxycycline, streptomycin or sulfonamides was not tested by disk diffusion or MicroScan.

### 3.4. Types of *stx* genes and subtypes. Other virulence genes, phylogenetic grouping, and sequence typing

The *stx* subtypes carried by isolated strains were distinguished by *in silico*-PCR, showing that six strains (15%) carried *stx*1 gene only: *stx1a* (*n* = 2) and *stx1d* (*n* = 4). Seventeen strains (43%) carried *stx*2 only: *stx2a* (*n* = 4), *stx2b* (*n* = 2), *stx2c* (*n* = 7), and *stx2d* (*n* = 4). Sixteen strains (41%) harbored combinations of *stx1* and *stx2* genes: *stx1a/stx2a* (*n* = 11), *stx1a/stx2c* (*n* = 2), and *stx1a/stx2d* (*n* = 3). Thirty-three strains (84.6% of all isolates) carried the subtypes associated with high pathogenic potency, *stx2a, stx2c*, or *stx2d.*

**Figure 2:**
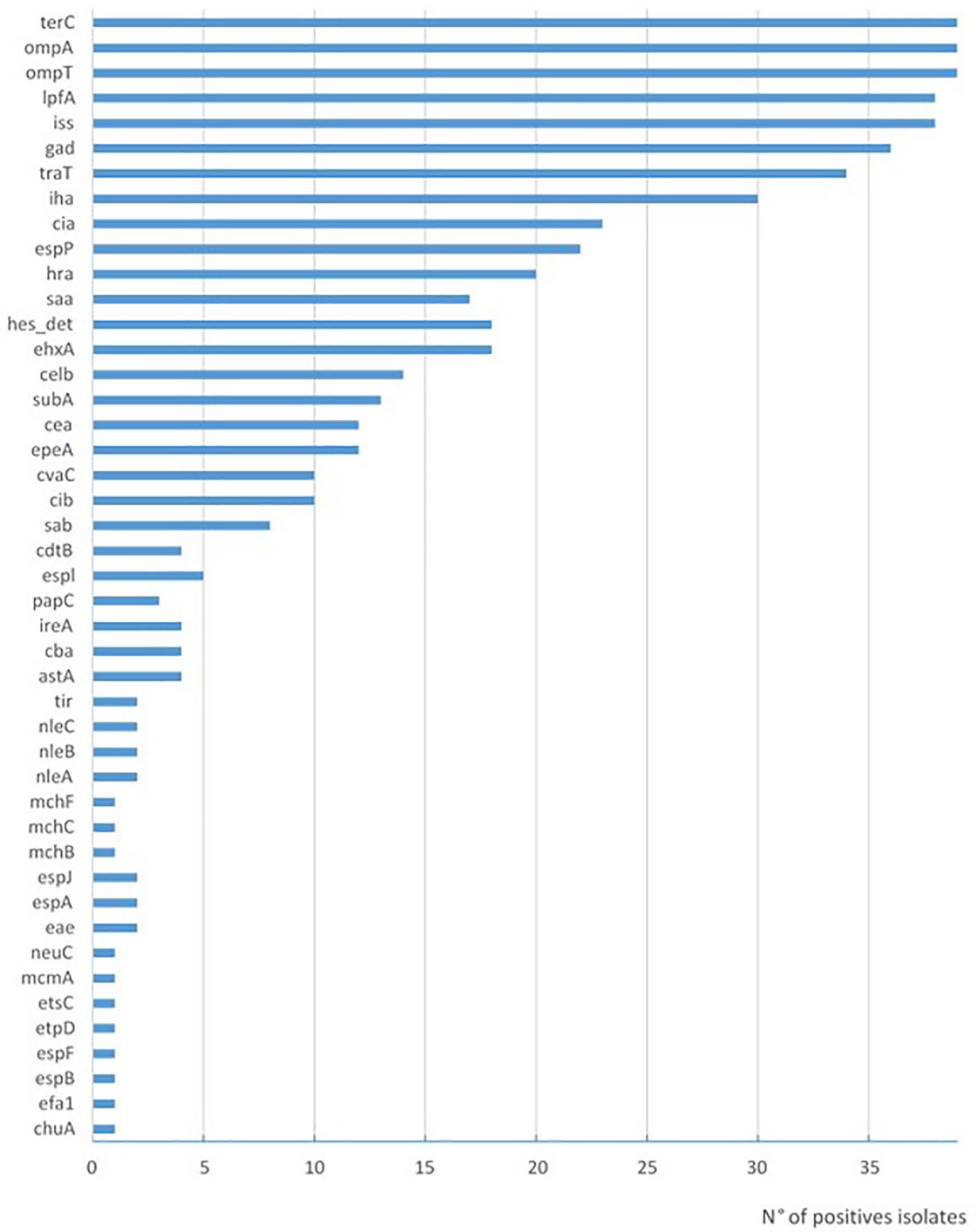
Prevalence of virulence genes in STEC strains isolated in Uruguay between 2018 and 2020 from chilled bovine carcasses.

Among the STEC strains investigated, a high variability of virulence gene profiles was identified (Table 2). One O130:H11 strain possessed the highest number of *E. coli* virulence-associated gene targets, with 21 genes detected. In addition to the *stx2a* gene, this strain presents genes associated with pathogenicity islands such as *iha*, and with plasmids *epeA* (pO113) and *espP* and *ehxA* (pO157). In relation to the amount of virulence factors, this strain is followed by three O174:H28 (20 virulence genes) and the O157:H7 with 19 virulence of the searched genes (Table 2). The virulence profile was examined in detail to detect hybrid strains searching for virulence markers founded in LEE-negative strains of animal or human origin, which were recognized in others pathotypes; *ipaH* as an indicator of enteroinvasive *E. coli* (EIEC), and *aggR*, *aat*, and *aaiC* as markers of enteroaggregative *E. coli* (EAEC). None of these genes was found in the 39 analyzed strains. The intimin coding gene *eae* was detected only in two strains, the O157:H7 and one O182:H25. The most prevalent genes found included those corresponding to adherence factors (Figure 2), such as *lpfA* coding long polar fimbriae (*n* = 38) and *iha* coding IrgA homolog adhesin (*n* = 30), outer membrane proteins *ompA* (*n* = 39) and *ompT* (*n* = 39); the serum resistance factor genes *iss* (*n* = 38) and *traT* (*n* = 34); the tellurite resistance gene *terC* (*n* = 39); the gene that codes for the glutamate decarboxylase *gad* (*n* = 36) and confers acid tolerance, and the bacteriocin colicin-Ia *cia* (*n* = 23). The iron utilization gene *chuA* was only present in the O157:H7 isolate (Table 2).

The majority (*n* = 38, 97.4%) of the isolates included in this study belonged to the phylogenetic group (Clermont type) B1, while the O157:H7 was classified as E.

The list of identified STs by MLST Finder 2.0 is shown in Table 2. Strains of the same serogroup and the same ST did not necessarily shared the same virulotype, evidencing again the genomic plasticity of these bacteria. For example, the three O174:H28 isolates of ST156 present three different virulence profiles, the six strains O130:H11 ST297 are distributed in four profiles, like the five strains of O22:H8 ST446 (Table 2).

### 3.5. Risk groups

**Figure 3:**
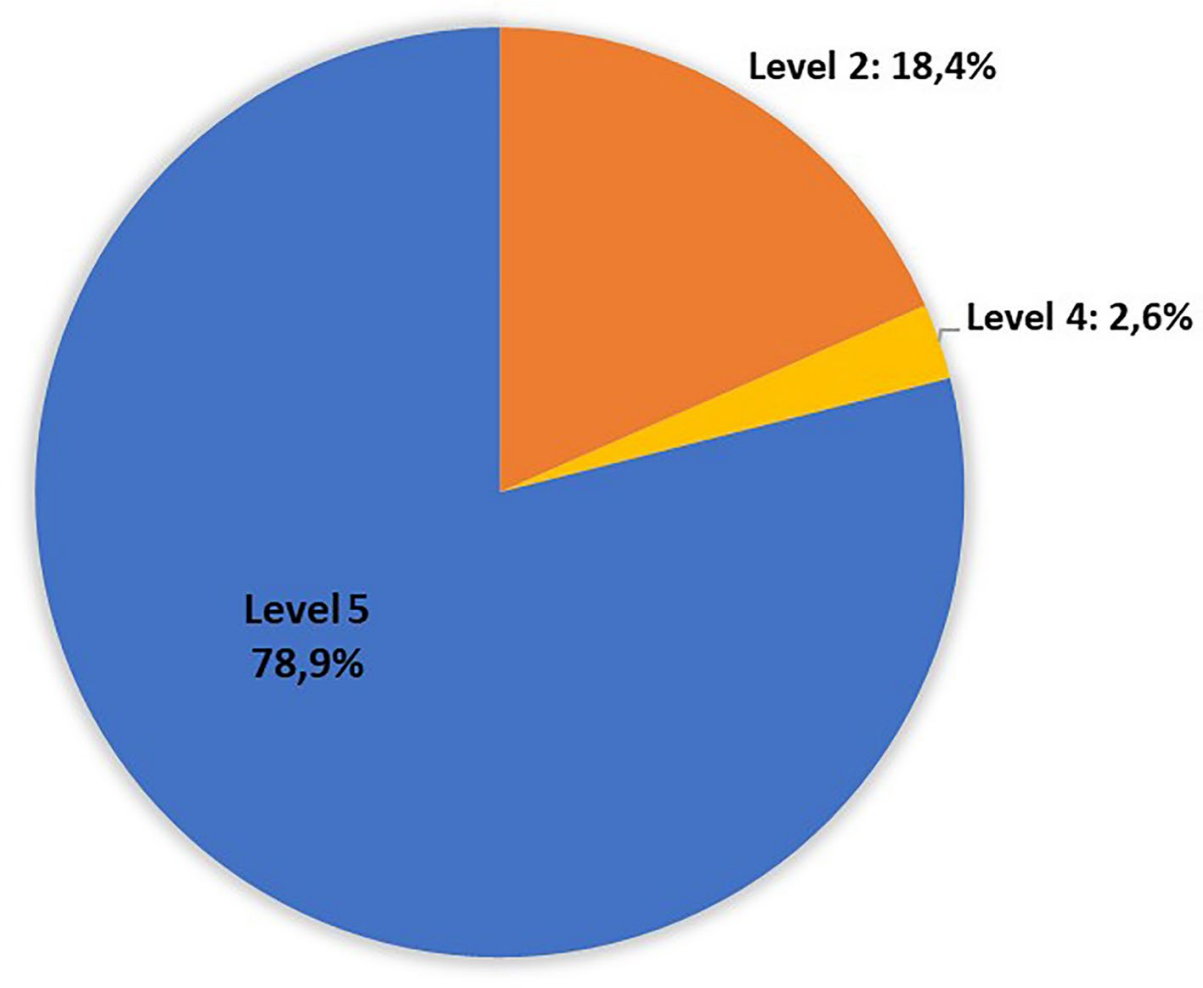
Distribution of non-O157 isolates recovered in Uruguay between 2018 and 2020 from chilled bovine carcasses in the risk levels defined by FAO/WHO: Level 1 “*stx2a + eae/aggR*”: 0 isolated; Level 2 “*stx2a*”: 7 isolates (O22:H8{2}, O113:H21{1}, O130:H11{2}, O174:H21{2}); Level 3”*stx2c + eae*”: none; Level 4 “*stx1a + ea*e”: 1 isolated (O182:H25); Level 5 “other *stx* subtypes”: 30 isolates (World Health Organization and Food and Agriculture Organization of the United Nations, 2018).

The distribution of the strains non-O157 in the risk groups indicated by the FAO/WHO, with the linked serogroups, were as follows: level 1 “*stx2a + eae/aggR*”: 0 isolated; Level 2 “*stx2a*”: 7 isolates (O22:H8{2}, O113:H21{1}, O130:H11{2}, O174:H21{2}); level 3”*stx2c + eae*”: none; level 4 “*stx1a + ea*e”: 1 isolated (O182:H25); level 5 “other *stx* subtypes”: 30 isolates (Figure 3).

## 4. Discussion

To our knowledge, this is the first local study on the presence of STEC in bovine carcasses ready to enter the production line, that includes the complete microbiological characteristics of the strains involved, such serotype, *stx* subtyping, sequence type, virulence and resistance genes.

In this work it was possible to estimate a 0.25% (CI 95 0–0.6%) prevalence of O157:H7 in chilled bovine carcasses (2 positives in 800 samples, both from the same internal supply slaughterhouse). Other published works reported prevalences of 2.7% in 258 refrigerated carcasses in Mexico (Varela-Hernández et al., 2007), 2.8% in 576 pre intervention carcasses on New Zealand (Stromberg et al., 2015), 3% in 132 carcasses in Ireland (Carney et al., 2006), 2.4% over 300 carcasses in Istanbul (Gun et al., 2003), 0.5% in a study carried out in Ethiopia with a similar sampling methodology as the used in our investigation (Abdissa et al., 2017). Also, a study carried out in export abattoirs in Argentina without post-slaughter interventions, reports a prevalence of 2.6% processed by immunomagnetic separation (Masana et al., 2011) and none O157:H7 was found in another study that analyzed pools of samples from 641 carcasses in an abattoir with a comprehensive HACCP design (Brusa et al., 2017). The lower prevalence found in our study may be due in part, to the fact that most STEC come from carcasses (500, 62%) of slaughterhouses that declared to apply some mitigation measures. Other types of study would be necessary to confirm or reject this hypothesis.

This study is the first experience in Uruguay working with this number of DNA sequences obtained by WGS of non-O157 STEC isolated from chilled bovine carcasses. Reports of these serotypes linked to human outbreaks have been increasing (Bettelheim and Goldwater, 2014). In Uruguay, in HUS cases with recovery of STEC, the serogroups O26, O111 and O145 predominate (Varela et al., 2008). In Argentina, with a robust surveillance system, non-O157 STEC are linked to 26% of HUS cases, with the most frequent serogroups being O145, O26, O121, and O103 (BIV 560 – Boletín integrado de Vigilancia N560 SE30, 2021). None of these serogroups were recovered in this work. This may be due to the low number of tested strains and some bias in their selection or to the fact that these strains are not as frequent in relation to other STEC in bovines. Another possible reason may be that we did not use immuno-concentration procedures nor chromogenic media that allow us to selectively identify them; or that its reservoir is not beef cattle. Also to that the STEC strains that produce HUS may come from other sources as indicated by EFSA and FAO/WHO (World Health Organization and Food and Agriculture Organization of the United Nations, 2018; Koutsoumanis et al., 2020).

Some of the serotypes analyzed corresponded to FAO/WHO level 2: O22:H8, O113:H21, O130:H11, and O174:H21. These serotypes have been previously isolated from patients with diarrhea, HC or HUS (Alonso et al., 2016; Commereuc et al., 2016; Oderiz et al., 2018). The knowledge of the origin of the STEC that cause severe disease in humans and their way of transmission continues to be a great question and therefore a challenge in studies related to these bacteria. Therefore, it would be of great importance to carry out continuous monitoring between strains isolated from humans and bovines as well as from other potential reservoirs to determine their relationship and eventual origin.

At the time of serotyping, we found some discordant results between both techniques used and some discrepancies could also be observed in the results obtained when assigning the serogroup by serology or when doing so using the Serotype Finder. The same occurred when Castro et al. compared PCR and WGS results to define *E. coli* serogroups (Castro et al., 2021). Some hypotheses to explain these discrepancies in our study are that we are dealing with new variants or new serogroups not yet described, and therefore not updated in the database used, or strains that cross-react with sera from other serogroups. Also, discrepant results may be due to sequence modifications in the O-antigen biosynthesis gene clusters (O-AGCs; Iguchi et al., 2015). More research could be done using new databases as they emerge.

Most of the strains (84%) carried *stx2* alone or associated with *stx1*, which has been shown to be related with more virulent lineages. At the same time, Stx2a is epidemiologically associated with an increase of the excretion levels of STEC O157 from cattle and of the transmission between animals. It could be because it is more rapidly produced than other Stx subtypes and limits cellular proliferation of bovine epithelial cells (Galarce et al., 2021). Only two isolates had the *eae* gene and none carried *aggR*, two of the most relevant virulence markers. Ninety-four point nine % were LEE-negative, but had different adhesin-encoding genes, including *lpfA*, *ehaA*, and *saa* (Table 2). LpfA is the major subunit of the fimbrial protein, able to bind fibronectin, laminin, and collagen IV (Farfan et al., 2011). EhaA is an autotransporter protein related with biofilm and cellular aggregation; while Saa is involved in adhesion to HEp-2 cells (Montero et al., 2019). Moreover, some recently acquired pathogenicity islands (PAI) could collaborate to its adhesion, such as LAA. Some genes are used as markers for PAI modules, for example *hes* is a marker for module I and *iha* and *lesP* are markers for module II (Vélez et al., 2020). Parts of this PAI was identified in 76.9% of the strains. Its acquisition is probably a recent evolutionary event in STEC (Montero et al., 2017), which could have contributed to the emergence of highly virulent LEE-negative strains. Other toxin-encoding genes detected were *ehxA* (46% of the isolates), *subA* (33%), and *cdtB* (10%), the last two have only been detected in LEE-negative strains. The plasmid-encoded enterohemolysin (EhxA) has the ability to form pores in several eukaryotic cell membranes and is widely distributed in STEC strains (Hua et al., 2021). SubAB integrates the AB5 toxin family. It is highly toxic for a range of cell types, induces vacuolization, and has a synergic effect with Stx2 in human glomerular endothelial cells damage, contributing to the development of HUS (Tsutsuki et al., 2022). Last, CDT causes irreversible G2/M arrest, death and inhibition of proliferation of human endothelial cells (Vélez et al., 2020). We must emphasize that no hybrid strain was found in this study.

An O99:H19 strain carried the *sul*2 genes; *tet(B); aph(3″)-Ib;* and *aph(6)-Id*; responsible for resistance to sulfonamides, tetracycline and streptomycin, respectively. Tetracycline resistance was confirmed with the MicroScan system, which yielded a MIC value >8 μg/ml. This occurs most frequently by the acquisition of genes that code for ribosomal protection proteins, efflux pumps, or also by enzymatic inactivation (Thaker et al., 2010; Gasparrini et al., 2020). The presence of these resistance and the *tetB* gene in STEC has been previously described in isolates from Chile and Irish cattle (Galarce et al., 2021). Many of the genes involved in antibiotic resistance are located in mobile elements, which emphasizes the fact that STEC can serve also as reservoirs for resistance genes that could be passed to other pathogenic or commensal microorganisms. In another STEC, the O120:H7, the gene *fosA7* (which confers resistance to fosfomycin) was found. The presence of this gene has been previously reported in *E. coli* and *Salmonella* spp. from animal and human origins (Gomi et al., 2017; Li et al., 2021), being an important concern in human health. Also, the presence of this gene in STEC from bovine feces and in a STEC O145:H25 isolated from an HUS case have been reported in our country (Mota et al., 2020; Umpiérrez et al., 2022).

The majority of STEC analyzed in this study (38/39) belonged to the B1 phylogroup, a finding similar to that previously reported in calves from Brazil (Coura et al., 2017). *Escherichia coli* belonging to the phylogroups A, B1, and D are the commensal and intestinal pathogenic strains, while the strains associated with extra-intestinal infection in humans belong to groups B2 and D. In animals, the predominance of B1 strains is informed, followed by A, B2 strains and, to a lesser extent, D (Ndegwa et al., 2022). The only O157:H7 strain included belonged to the phylogroup E. Although the *E. coli* strains of the found phylogroups (B1 and E) are associated with diseases in humans, it is very difficult to establish a link between phylogenetic groups and severity of infection in humans.

The ST297 was the most frequently detected. Strains of this ST were detected worldwide from non-human and human hosts and are associated with illness in humans and animals. This ST was predicted from genomes of STEC strains isolated from humans and beef in Chile and has been identified as the predominant lineage in cattle from Sweden (Carter et al., 2016), and in cattle, milk, pigs and water from farms in China (Peng et al., 2019). We also identified 5 isolates of ST446 (O22:H8), this serogroup was described to have a protector effect versus O157:H7 competing at the bovine recto-anal junction, making non-O157 carrying-calves less susceptible to O157:H7 colonization (Martorelli et al., 2017). Also the ST443, found in this study, was identified in cattle and beef samples from Chile and Uruguay before. The O157:H7 ST11 has been described as the principal clone identified from human infections in Argentina, Brazil, Paraguay and Uruguay and its expansion can be responsible for causing severe disease, as HUS (Vélez et al., 2020).

A quantitative risk assessment carried out in Argentine abattoirs that applied HACCP published in 2020, concludes that only 10% of HUS cases would be linked to beef consumption. In that work it is also inferred that the food with the highest risk is ground beef and that no case of HUS would be expected from the consumption of beef cuts (Brusa et al., 2020). It would be convenient for a similar study to be carried out in Uruguay.

It is interesting to see that strains of the same serogroup, ST and Clermont type were recovered from different establishments located in different regions. This may be due to the fact that several slaughterhouses are often supplied by the same animal breeders favoring the distribution of identical STEC strains to different establishments. Another particular case is that of the U14 and U37 strains, both are O130:H11 that come from the same slaughterhouse from different carcasses and on the same date but showed differences in their virulence profile. This may be due to the instability (by loss or gain) of genes encoding different virulence attributes and highlights the plasticity of the *E. coli* genome.

## 5. Conclusion

This is the first local work carried out with the aim of studying the STEC present in bovine carcasses, without performing any serogroup pre-selection, as is generally done in meat industry and control agencies. The idea was to have a notion of the diversity of the circulating STEC strains, their associated virulence factors, their resistance profile and estimate the potencial risk to produce severe human diseases.

Thus, 39 isolates were studied; one O157:H7 and 38 non-O157 corresponding to 19 different O-groups, resulting the most frequent serotypes: O130:H11 (six strains), O174:H28(5), and O22:H8 (5), all of them previously isolated from humans and animals/derived foods in other countries.

Using WGS it was possible to study the presence of multiple virulence factors, resistance genes and determine the sequence type and phylogroup of this set of STEC. This tool is still very expensive for us and is not routinely used for epidemiological surveillance nor to characterize this group of pathogens in our country. However, this work gave us the possibility to show its potential and obtain a lot of information that would have been very laborious to achieve with traditional PCR techniques.

Beyond the bias in the selection of the STEC analyzed, the obtained data suggest that cattle constitute a local reservoir of non-O157 serotypes associated with severe diseases in humans. In that sense, although most of the isolates studied belonged to the least risky FAO/WHO levels, some of the serotypes analyzed corresponding to level 2 (O22:H8, O113:H21, O130:H11, and O174:H21) were previously isolated in other countries from patients with diarrhea, HC or HUS. Other studies are needed to define the local participation of these serotypes in these human pathologies.

Anyway, the information gathered in the present study help to a better estimation of the risk from non-O157 STEC in the beef industry of Uruguay and American region. Since other vehicles can be involved in the local spread of STEC, studies similar to this, incorporating analysis by WGS, should be performed including a larger number of strains recovered form beef, other foods and environment to have a better epidemiological link to cases of human STEC infections.

## Data availability statement

The data presented in the study are deposited in the NCBI repository, accession number of each strain is detailed on the supplementary files: https://dataview.ncbi.nlm.nih.gov/object/PRJNA914614?reviewer=4qmj8hptqcfl104prsud5h1ujs.

## Author contributions

PM, IM, SL, GL, and GV contributed to conception and design of the study. PM performed the most of the analytical part and AN was the responsible for the serological serotyping of the strains. All authors contributed to manuscript revision, read, and approved the submitted version.

## Funding

This work was supported by Agencia Nacional de Investigación e Innovación (ANII), Fondo Sectorial Innovagro – Inocuidad, Grant: FSA_I_2017_1_140224.

## Conflict of interest

The authors declare that the research was conducted in the absence of any commercial or financial relationships that could be construed as a potential conflict of interest.

## Publisher’s note

All claims expressed in this article are solely those of the authors and do not necessarily represent those of their affiliated organizations, or those of the publisher, the editors and the reviewers. Any product that may be evaluated in this article, or claim that may be made by its manufacturer, is not guaranteed or endorsed by the publisher.
